# New approaches to disinfection of thermolabile medical devices using an indirect method with cold atmospheric plasma-aerosol

**DOI:** 10.1038/s41598-025-03364-2

**Published:** 2025-06-02

**Authors:** Tom Schaal, Ulrich Schmelz, Frank-Albert Pitten, Tim Tischendorf

**Affiliations:** 1 Faculty of Health and Healthcare Sciences, University of Applied Sciences Zwickau, Zwickau, Germany; 2 University of Fulda, Fulda, Germany; 3 IKI Institute for Hospital Hygiene & Infection Control GmbH, Gießen, Germany

**Keywords:** Plasma physics, Medical research, Risk factors, Public health

## Abstract

Cold atmospheric plasma-aerosol (CAP-A) offers a promising alternative to conventional sterilisation and disinfection methods, which are often unsuitable for thermolabile medical devices due to high temperatures, toxic chemicals or radiation. CAP-A efficiently inactivates microorganisms and viruses without compromising the material integrity. Given the ongoing risk of infection associated with ultrasound probes and other delicate diagnostic instruments, this study investigates whether an indirect CAP-A method can meet all requirements for effective and safe disinfection of thermolabile medical devices. The disinfection of thermolabile medical devices was carried out in a container saturated with indirect CAP-A. A transvaginal ultrasound probe was used as a reference product. The study involved six test organisms, with five measurements taken at six different measurement points. The study showed that *Enterococcus hirae* (mean logarithmic reduction factor (LRF) > 6.23), *Staphylococcus aureus* (mean LRF > 6.51), and *Enterococcus faecium* (mean LRF > 6.16) demonstrated a germ reduction of > 99.9999%. For *Pseudomonas aeruginosa* (mean LRF > 5.40) and *Escherichia coli* (mean LRF > 5.29), a germ reduction of > 99.999% was achieved, and for *Candida albicans* (mean LRF > 4.95) and *Clostridioides difficile* (mean LRF > 4.62), a germ reduction of > 99.99% was demonstrated. The log reduction demonstrates a complete inactivation of the six tested microorganisms. The initially defined requirements for an effective disinfection process for thermolabile medical devices were met in the CAP-A method. Regarding highly tenacious microorganisms, such as *Clostridioides difficile*, the method of CAP-A proved effective, superior to alcohol-based methods, and with no resistance development observed. Its efficacy is otherwise only known in corrosive chemicals, such as hydrogen peroxide, chlorine, and chlorine dioxide. However, these chemicals have corrosive-oxidative effects on the surfaces to be disinfected and are critical in terms of market launch and hazardous material classification. Therefore, the method of CAP-A, provides an effective, material-friendly alternative.

## Introduction

The established sterilization and disinfection methods that rely on high temperatures, radioactive radiation, or the use of highly reactive and often toxic chemicals are not applicable to many products and fields in hygiene and medicine. The indirect cold atmospheric plasma-aerosol (CAP-A) method offers a promising alternative, as it has been shown to not only inactivate microorganisms, viruses and yeasts, but also to inactivate forming of biofilms. Additionally, there have not been reported cases of microorganisms developing resistance to cold atmospheric plasma (CAP). Further CAP is effective against multiresistant microorganisms^1–5^. With regard to high-quality, thermolabile medical devices in imaging, challenges arise that yet have not been sufficiently solved. Both optical and ultrasound instruments, such as gastroscopes or vaginal ultrasound probes, contain complex optical and electronic components that practically exclude thermal disinfection^6–8^.

To be reused, medical products must undergo hygienic reprocessing. Only thereafter, they can be used on multiple patients without a significantly increased risk of infection. Many instruments can easily be reprocessed using the standard European method for disinfecting medical devices—thermal or mechanical-thermal disinfection at 93 °C for at least three minutes (e.g. scissors, needle holders, scalpels, forceps, etc.)^9^. The present study focuses on the more fragile devices, e.g. vaginal ultrasound probes. Transvaginal ultrasound probes are routinely used to examine suspected gynecological conditions, to evaluate complications in early and late pregnancy, as well as for primary prevention in the context of tumor screening^10^. They are considered semicritical devices due to their contact with intact mucous membranes and should be used with a suitable probe cover and subjected to high-level disinfection (HLD) after use, as infectious bacteria and viruses can persist on probes, probe covers and ultrasound gel^11–13^. If barrier protection is inadequate and hygiene of the Sonic head is insufficient, contamination after disinfection is likely^14^. Several pathogens have been identified on probes after disinfection. These include methicillin-resistant Staphylococcus aureus, human papillomavirus (HPV), and human immunodeficiency virus (HIV)^15–18^. The concerning HPV types 16 and 18 are common causes of cervical cancer and are highly resistant to decontamination^17–19^. Factors such as a lack of awareness of the transmission risk, inadequate knowledge or training of staff, and lack of prioritization of infection prevention contribute to noncompliance with infection prevention^20^.

**Fig. 1:**
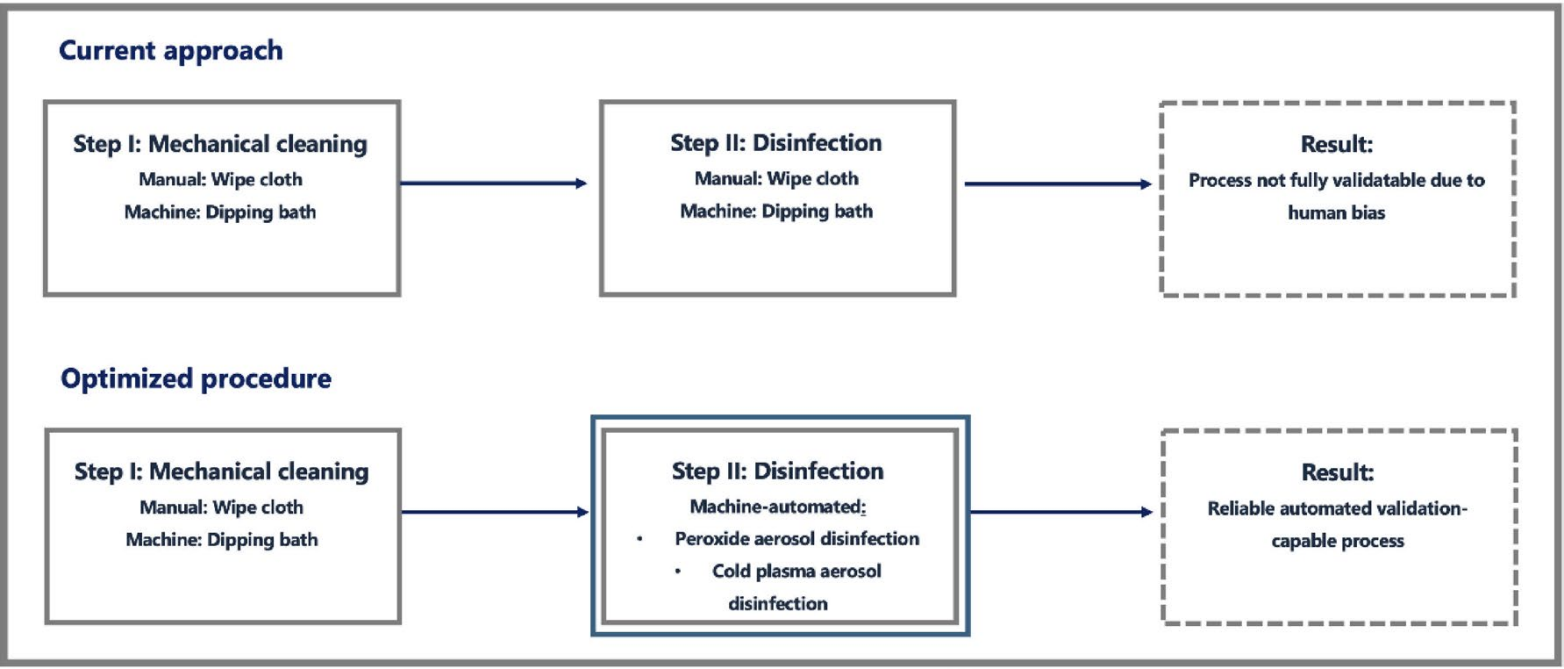
Reprocessing of thermolabile medical devices for diagnostic imaging. Current approach and optimized approach using indirect Cold Atmospheric Plasma-Aerosol (CAP-A). Source: Own illustration.

The vast majority of ultrasound probes and many other thermolabile devices cannot be thermally disinfected. For many years, soak bath or wipe disinfection have been the only options. However, the manual nature of wipe disinfection prevents standardization and validation according to current quality management standards due to “human bias”. Therefore, the reproducible disinfection of ultrasound probes in the current approach remains unsolved (Fig. 1).

In total, there is a significant risk of infection. A study by Carrico et al. found that of 358 US healthcare facilities, 20% reported errors in the processing of ultrasound probes, with the majority of these cases occurring in obstetrics and gynecology, and in emergency departments^21,22^.

It is not acceptable that this risk is caused by a purely primary preventive examination, usually in the context of tumor diagnostics. Handling thermolabile medical devices in imaging on the basis of the CAP-A method could result in a significant improvement in hygienic safety.

Both the Robert Koch Institute in Germany and the EU health authorities strongly recommend the provision of a reproducible and validatable reprocessing of invasively used ultrasound probes^8^. In the EU, a guideline-compliant reprocessing of ultrasound probes is mainly carried out using hydrogen peroxide. To do this, hydrogen peroxide is brought into contact with the ultrasound probe in the form of an aerosol that is as fine as possible. Such disinfection is reproducible and validatable, as the time and hydrogen peroxide density in the aerosol can be precisely set. A sufficient efficacy is achieved, making this process an important milestone in terms of more infectiological safety. However, while this method is effective in reducing germs due to its sufficient germ reduction of a defined initial germ load by more than five decimal places (10^−5^), there are still certain points that should be optimized to make this process even more widely applicable and to almost completely exclude the transmission of germs by ultrasound probes.

Recent outbreaks of viral diseases have emphasized the need for effective virus disinfection techniques^23^. Indirect CAP-A emerges as a particularly promising approach due to its ability to quickly and effectively inactivate microorganisms. The inactivation is based on the conditioning of the aerosol through generation of electrical fields, charged particles, reactive oxygen and nitrogen species, and ultraviolet radiation, without significantly increasing the temperature of the treated substrate^24^. For example, effective disinfection of surfaces in ambulances using the method of CAP, as well as the superiority of hand disinfection using the further development method of CAP-A over alcohol-based disinfection methods, has been demonstrated^4,5^. To meet the specific requirements for thermolabile instruments like ultrasound probes using a new approach, the process must achieve in average over five log levels of germ reduction, be reproducible, validatable, and automatable, not cause surface damage or impair electronics, have minimal environmental impact, and be cost-effective with a temporarily present active ingredient.

This study aims to answer the question of whether an indirect cold atmospheric plasma based method can meet all the requirements for the disinfection of thermolabile medical devices.

## Methods

### Description of the applied technique: the indirect Cold Atmospheric Plasma-Aerosol (CAP-A) method

**Fig. 2:**
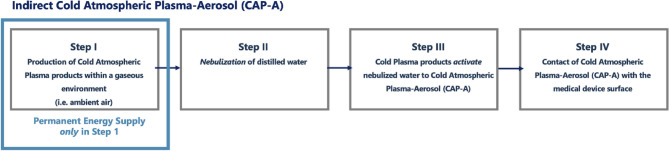
Schematic overview of the indirect Cold Atmospheric Plasma-Aerosol (CAP-A) process applied in this study. Source: Own illustration.

**Fig. 3:**
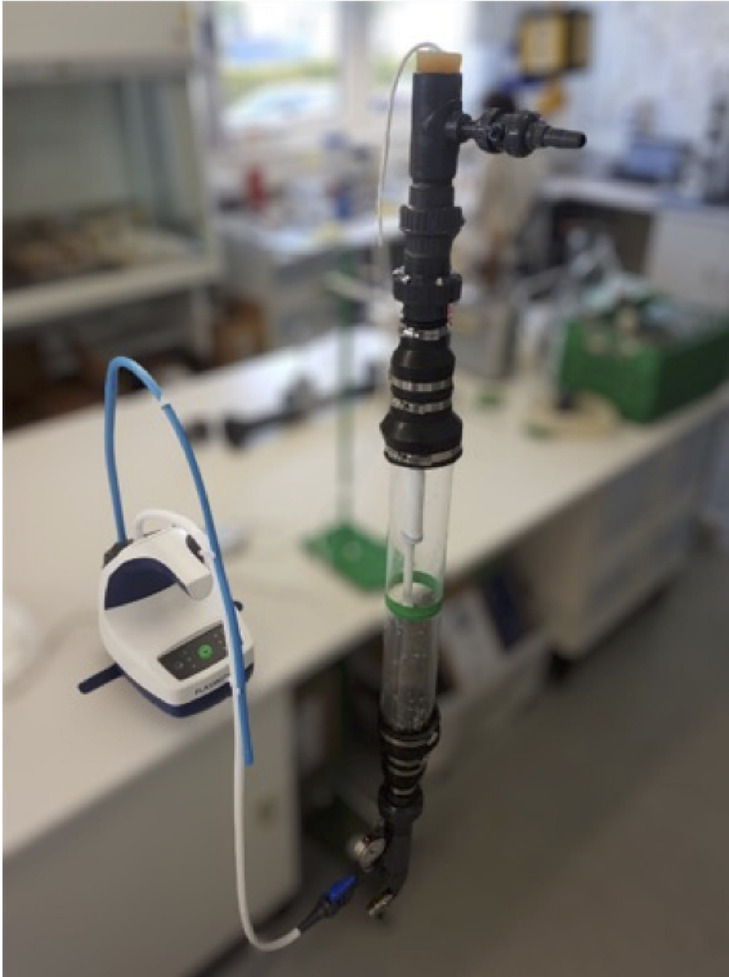
Set-up: exposure of the ultrasound probes in an exposure tube. Source: Own illustration.

**Fig. 4:**
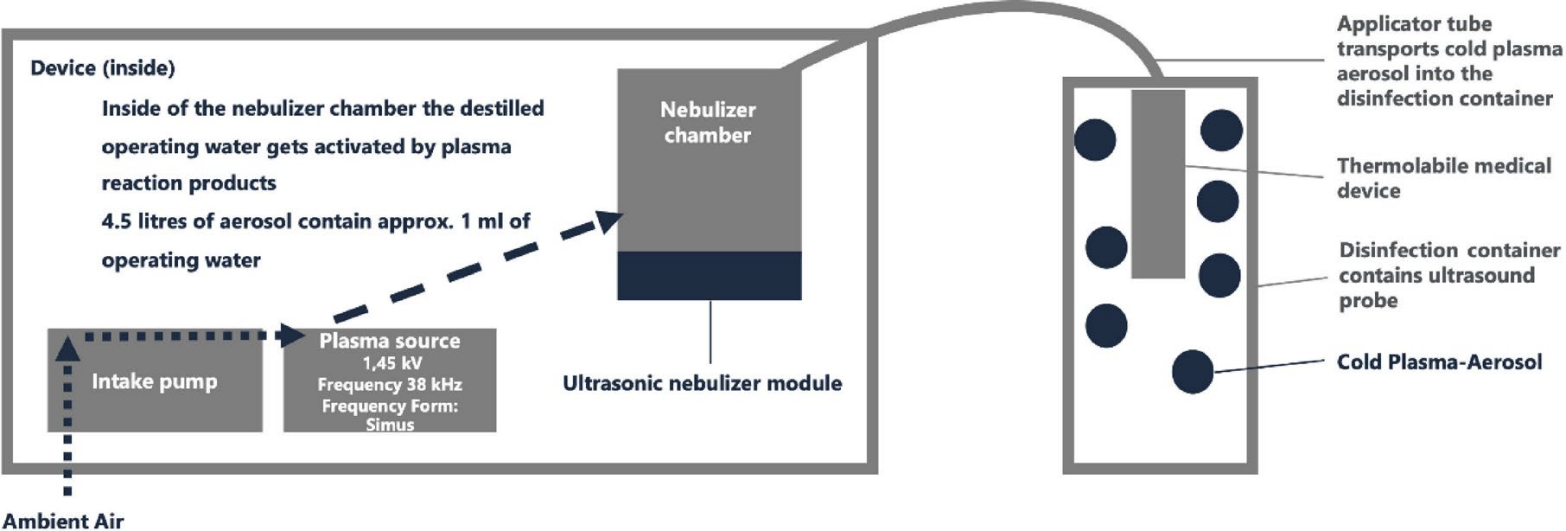
Set-up: exposure of the ultrasound probes in an exposure tube (graphical image). Source: Own illustration.

The applied process represents the above mentioned (CAP-A) method. The device "PLASMO®HEAL PRO" produces plasma reaction products, using ambient air and electricity within the device’s cold plasma source^25,26^. As a result of the reaction of oxygen and water vapor in ambient air, hydroxyl radicals as part of Reactive Oxygen Species (ROS) are primarily formed in the atmospheric cold plasma reaction^27^. The plasma reaction products then enrich an aerosol that is produces separately in the device. The air enriched with hydroxyl radicals is combined with water droplets from an ultrasonic atomizer. The resulting cold plasma-activated aerosol via ROS profits from an electrostatic charge resulting in a higher performance of the subsequent solvent and dilution effect^28^. The conditioned aerosol is then applied to the intended area of disinfection via a tube^25,26^ (Figs. 3 and 4). A schematic illustration of the CAP-A process is shown in Fig. 2. The figure summarizes the four main steps of the process (Fig. 2).

In the CAP-A system, reactive plasma species are first generated from ambient air using a dielectrically impeded discharge (Step I, Fig. 2). In parallel, distilled water is nebulized into fine droplets using an ultrasonic nebulizer (Step II, Fig. 2). These droplets are enriched with the reactive plasma species in a mixing chamber, resulting in a cold plasma activated aerosol (CAP-A) (Step III, Fig. 2). The aerosol is then continuously delivered through a flexible tube into an exposure tube. The medical device to be disinfected (e.g. a vaginal ultrasound probe) is placed inside this exposure tube, ensuring full surface contact with the aerosol under ambient conditions (Step IV, Fig. 2). The probe is exposed to the aerosol for 15 min, with a continuous flow to ensure saturation throughout the tube. The test device has a control/indicator light. This device ensures constant operating conditions for the device’s plasma source. It measures the energy supply in mA.

During the test run, it is considered desirable that no unwanted by-products such as ozone or nitrogen oxides are produced during the indirect atmospheric cold plasma reaction. Parallel physical experiments at different voltages have shown that the plasma cell at 1.75 kV produces no nitrogen oxides and only traces of ozone. Nitrogen oxide emission starts at a voltage of about 5 kV. Ozone emission starts significantly at a voltage of about 3 kV. By limiting the voltage of the dielectrically impeded discharge to 1.75 kV (and by suitable electrode materials in conjunction with the optimization of electrode surfaces), it can be achieved that ozone formation is insignificant, and at the same time, no nitrogen oxides are formed. The underlying device, PLASMO®HEAL PRO, demonstrated ozone concentrations of 42.7 ppb in a controlled laboratory environment after four cycles of 3-min treatment within one hour. This value is significantly below the limit value of 50 ppb required by the IEC 60335-2-65 standard. The test was conducted in a closed environment to prevent unwanted air exchange and ensure that no exogen variables influenced ozone concentrations during operation. In clinical settings where the CAP-A process takes place in closed containers (Fig. 3) and within seetings including airflow dynamics, the safety of the method is further enhanced as this minimizes the potential for ozone exposition to the operator.

### Application of Cold Atmospheric Plasma–Aerosol (CAP-A) for disinfection of thermolabile medical devices

The disinfection of thermolabile medical devices is carried out in a container that is saturated with the aerosol from the plasma disinfection device. For this investigation, a vaginal ultrasound probe (GE IC9-RS micro-convex vaginal ultrasound probe (H48691PJ), GE Healthcare Austria GmbH & Co OG) is used as a reference product. This medical device is representative of other thermolabile medical devices, such as gastroscopes, bronchoscopes, or colonoscopes.

For the laboratory scale, the exposure of ultrasound probes takes place in an exposure tube that is continuously flowed with activated aerosol from the plasma disinfection device (Figs. 3 and 4). A PVC-U pipe DN 100 with a length of 1 m is used as an exposure tube, which encompasses a volume of approx. 7.5 l. The tube is exposed 15 min to PLASMO®HEAL PROs aerosol in order to ensure that the ultrasound probe in the tube is located in a saturated aerosol environment. The device maintains a continuous flow through the exposure tube. The exposure tube is positioned vertically for this purpose. At the lower end of the device, the described aerosol is introduced and exits at the upper point into the atmosphere. Exposure takes place at atmospheric pressure and room temperature around 21 °C.

### Quantitative suspension test with application-oriented test specimens (vaginal ultrasound probes) to determine microbicidal efficacy

**Fig. 5:**
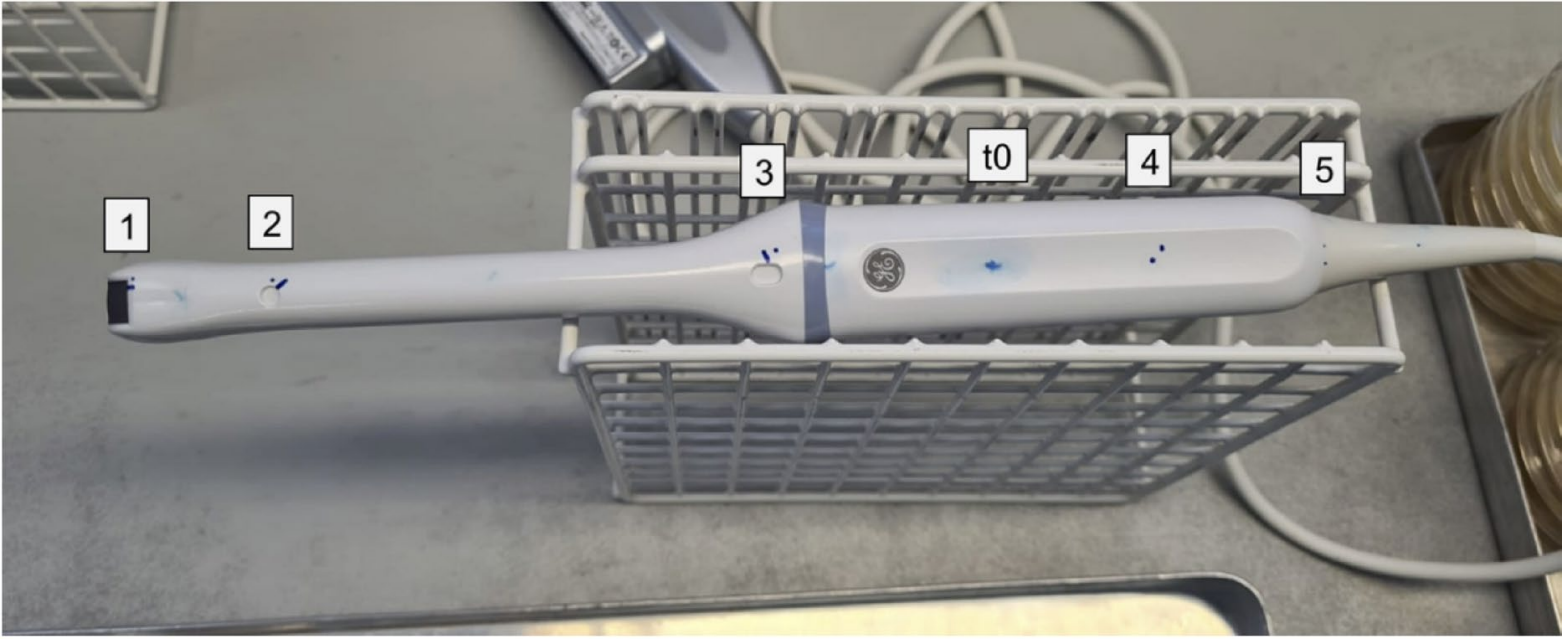
Contamination of the probe. Contamination points one to five are test points whose bacterial count density is determined after exposure. At contamination point t0, the reference value of the initial bacterial count is taken for comparison after the test bacterial contamination and before exposure. Source: Own illustration.

The ultrasound probe is suspended in the exposure tube as a test specimen, on which test germ contaminations have been previously applied. The test germ contamination is applied to an area of approx. 1 cm^2^ of the ultrasound probe using a cotton swab. Six contamination points are applied to the ultrasound probe in this manner (Fig. 5).

The probe is fixed in its position in the center of the exposure tube by means of a perforated and sagittally cut silicone stopper on the cable. After inserting the ultrasound probe into the exposure tube, the plasma disinfection device is activated. The probe is exposed to the plasma-activated aerosol for 15 min under continuous flow conditions. Then, after turning off the test apparatus, the ultrasound probe is immediately removed, and the germ density at the five test points is determined after exposure to the disinfection process.

## Laboratory execution

### Material and methodology of microbiological analysis

An in vitro test of the germ reduction performance against seven normatively defined microorganisms was carried out in accordance with DIN spec. 91315:2014^29^. The identity of the test organism was verified by Gram staining in conjunction with transmitted light microscopy and by determining the metabolic activity of the test organism in relation to various substrates. Additionally, the test organism was identified as indole-forming from tryptophan (indole-positive). The universal medium Caso agar in 60 mm Petri dishes was used as the culture medium for the sample inocula.

For each of the mentioned test organisms, an isolated exposure was performed. The test organisms were first cultured on TSA plates after identity confirmation. After incubation (conditions and time see Table 1), the microorganisms grown on the nutrient agar in the Petri dishes were brought into suspension with sterile glass beads by overlaying with NaCl 0.9% solution and by radial shaking.

**Table 1:** Microorganisms used for the test in accordance with DIN spec. 91315.

Microorganisms	Germ identification	Culture media with identification number	Incubation time and temperature
*Staphylococcus aureus*	ATCC 6538	Mannitol-salt-agar No.1050	48 h/36 °C
*Escherichia coli*	NCTC 10538	Colichromer-agar No. 4028	48 h/36 °C
*Pseudomonas aeruginosa*	ATCC 15442	Cetrimid-agar No. 4025	48 h/36 °C
*Candida albicans*	ATCC 10231	Sabouraud-agar 2% No. 4130^1^	72 h/28 °C
*Enterococcus faecium*	ATCC 6057	Slanetz Bartley Agar No 5241	48 h/44 °C
*Enterococcus hirae*	ATCC 10541	Slanetz Bartley Agar No 5241	48 h/44 °C
*Clostridioides difficile R027*	NCTC 13366	BHIYT-L Agar	36 ± 1 °C, 5 days, anaerobic

^1^The microbiological culture media for this analysis were obtained from Dr Möller & Schmelz, Göttingen, Germany.; ATCC = American Type Culture Collection. NCTC = National Collection of Type Cultures.

The suspension must have a turbidity of McFarland Standard > 7, which corresponds to a bacterial concentration of approximately (1.3 to 5.3) * > 10^7^ CFU/ml, depending on the microorganism, as shown in Table 2. The germ count density was determined in parallel by a dilution series in the laboratory (Table 2).

**Table 2:** Microbial suspensions for the different test germs.

Microorganism	Suspension (X * 10^7^ CFU/ml)
Enterococcus hirae*	4.2
Staphylococcus aureus*	5.3
Candida albicans**	6.1
Pseudomonas aeruginosa*	4.8
Escherichia coli K 12*	1.3
Enterococcus faecium*	3.2
Clostridioides difficile***	2.2

*Bacteria. **Yeast. ***in form of bacterial endospores CFU/ml = colony-forming units per milliliter.

A vaginal ultrasound probe was contaminated with five µl of test suspension of one of the seven discussed microorganisms at each of six test points mentioned above (Fig. 5). After drying in a clean-room bench a swab was soaked in diluent (NaCl 0,9%) and the spot of the control (1 cm^2^) was rubbed. The swab was washed out in 5 ml diluent. The procedure was repeated with a dry swab which was washed out in the same five ml diluent. From there ten-fold dilutions were prepared and one ml of each dilution was spread out on agar plates (pour plates in case of C. difficile).

After exposure to the disinfection process, germ density is measured at five test points. To assess contamination levels before and after CAP-A treatment, the designated area on the ultrasound probe is wiped with a sterile swab soaked in standardized hard water. The swab is then placed in a test tube containing 10 ml of 0.9% NaCl solution and mixed for three minutes using a Vortex® shaker. A tenfold serial dilution is prepared from the initial suspension (10^0^) up to 10^−5^. From each dilution step, 100 µl is applied to the appropriate nutrient medium and evenly distributed using a sterile Drigalski spatula. The disinfection of thermosensitive medical devices was carried out in a laboratory environment under controlled conditions. The experimental protocols were approved by Dr. Schmelz GmbH Malsfeld and Umwelthygiene Marburg GmbH & Co. KG in October 2024.

### Evaluation of the laboratory findings

After incubation (see Table 1), the colony count of the dilution stage that shows 10–100 colonies on the nutrient medium is determined. Subsequently, the colony count per surface equivalent of 1 cm^2^ (at the respective contamination point) is calculated from colony count, dilution stage, inoculation volume (100 µl) and initial volume ten ml (in which the transfer swab was rinsed). Afterwards, the colony count is converted into the decimal logarithm, making it proportionally comparable to a first-order reaction kinetics (which is present in the inactivation of microorganisms). Therefore, the logarithmic reduction factor (LRF) can be calculated by subtracting the logarithm of the initial germ count from the logarithm of the germ count after disinfection.

$${\text{LRF }} = {\text{ log }}\left( {\text{N initial}} \right) \, - {\text{ log }}\left( {{\text{N after Exp}}.} \right)$$

According to the recommendations of the VAH and other European hospital hygiene societies, disinfection processes regarding bacteria must achieve a reduction of more than five log levels (LRF)^30^. This results in a 100,000-fold reduction in germ count, achieving the state of "absence of infectious potential", called “asepsis”.

## Results

The aim of the study was to investigate the ability of the CAP-A process to meet the requirements for disinfection of thermolabile medical devices. The antimicrobial properties of the process are confirmed by different levels of testing as shown in Table 3. The study demonstrates effective reduction performance in terms of killing various microorganisms with log reduction factors ranging from 4.62 to 6.51. The log reduction factors obtained represent a maximum microbial effect in the form of complete inactivation of all inoculated test microorganisms. The results showed that *Enterococcus hirae* (mean LRF > 6.23), *Staphylococcus aureus* (mean LRF > 6.51), and *Enterococcus faecium* (mean LRF > 6.16) had a germ reduction of over 99.9999%. For *Pseudomonas aeruginosa* (mean LRF > 5.40) and *Escherichia coli* (mean LRF > 5.29), a germ reduction of 99.999% was shown, while Candida albicans (mean LRF > 4.95) and *Clostridioides difficile* (mean LRF > 4.62) had a germ reduction of 99.99%.

**Table 3:** Results of the CAP-A method for the disinfection of heat-sensitive medical devices.

Microorganism	Initial number of germs	Test sample (Log CFU per Area of contamination after exposure)					Mean [1:5]	LRF	Mean LRF
	log (CFU) [initial]	1	2	3	4	5			
Enterococcus hirae*	6.45	< 1	< 1	< 1	< 1	< 1	< 1	> 6.45	
	6.06	< 1	< 1	< 1	< 1	< 1	< 1	> 6.06	
	6.28	< 1	< 1	< 1	< 1	< 1	< 1	> 6.28	
	5.96	< 1	< 1	< 1	< 1	< 1	< 1	> 5.96	
	6.38	< 1	< 1	< 1	< 1	< 1	< 1	> 6.38	
	x̄ = 6.23								> 6.23
Staphylococcus aureus*	6.85	< 1	< 1	< 1	< 1	< 1	< 1	> 6.85	
	6.18	< 1	< 1	< 1	< 1	< 1	< 1	> 6.18	
	6.32	< 1	< 1	< 1	< 1	< 1	< 1	> 6.32	
	7.40	< 1	< 1	< 1	< 1	< 1	< 1	> 7.40	
	5.78	< 1	< 1	< 1	< 1	< 1	< 1	> 5.78	
	x̄ = 6.51								> 6.51
Candida albicans**	5.05	< 1	< 1	< 1	< 1	< 1	< 1	> 5.05	
	4.63	< 1	< 1	< 1	< 1	< 1	< 1	> 4.63	
	5.21	< 1	< 1	< 1	< 1	< 1	< 1	> 5.21	
	4.78	< 1	< 1	< 1	< 1	< 1	< 1	> 4.78	
	5.10	< 1	< 1	< 1	< 1	< 1	< 1	> 5.10	
	x̄ = 4.95								> 4.95
Pseudomonas aeruginosa*	5.72	< 1	< 1	< 1	< 1	< 1	< 1	> 5.72	
	5.30	< 1	< 1	< 1	< 1	< 1	< 1	> 5.30	
	5.15	< 1	< 1	< 1	< 1	< 1	< 1	> 5.15	
	5.65	< 1	< 1	< 1	< 1	< 1	< 1	> 5.65	
	5.18	< 1	< 1	< 1	< 1	< 1	< 1	> 5.18	
	x̄ = 5.40								> 5.40
Escherichia coli K 12*	5.09	< 1	< 1	< 1	< 1	< 1	< 1	> 5.09	
	5.85	< 1	< 1	< 1	< 1	< 1	< 1	> 5.85	
	4.98	< 1	< 1	< 1	< 1	< 1	< 1	> 4.98	
	5.20	< 1	< 1	< 1	< 1	< 1	< 1	> 5.20	
	5.35	< 1	< 1	< 1	< 1	< 1	< 1	> 5.35	
	x̄ = 5.29								> 5.29
Enterococcus faecium*	6.08	< 1	< 1	< 1	< 1	< 1	< 1	> 6.08	
	5.98	< 1	< 1	< 1	< 1	< 1	< 1	> 5.98	
	6.23	< 1	< 1	< 1	< 1	< 1	< 1	> 6.23	
	6.18	< 1	< 1	< 1	< 1	< 1	< 1	> 6.18	
	6.35	< 1	< 1	< 1	< 1	< 1	< 1	> 6.35	
	x̄ = 6.16								> 6.16
Clostridioides difficile***	4.81	< 1	< 1	< 1	< 1	< 1	< 1	> 4.81	
	4.62	< 1	< 1	< 1	< 1	< 1	< 1	> 4.62	
	4.39	< 1	< 1	< 1	< 1	< 1	< 1	> 4.39	
	4.75	< 1	< 1	< 1	< 1	< 1	< 1	> 4.75	
	4.53	< 1	< 1	< 1	< 1	< 1	< 1	> 4.53	
	x̄ = 4.62								> 4.62

CFU can only be whole natural numbers, which is why < 1 is the smallest possible unit that can be counted on the culture medium. *Bacteria. **Yeast. ***in form of bacterial endospores. Exposure time 15 min. CfU = Colony forming Unit; x̄ = Mean initial contamination; LRF = log reduction factors.

**Fig. 6:**
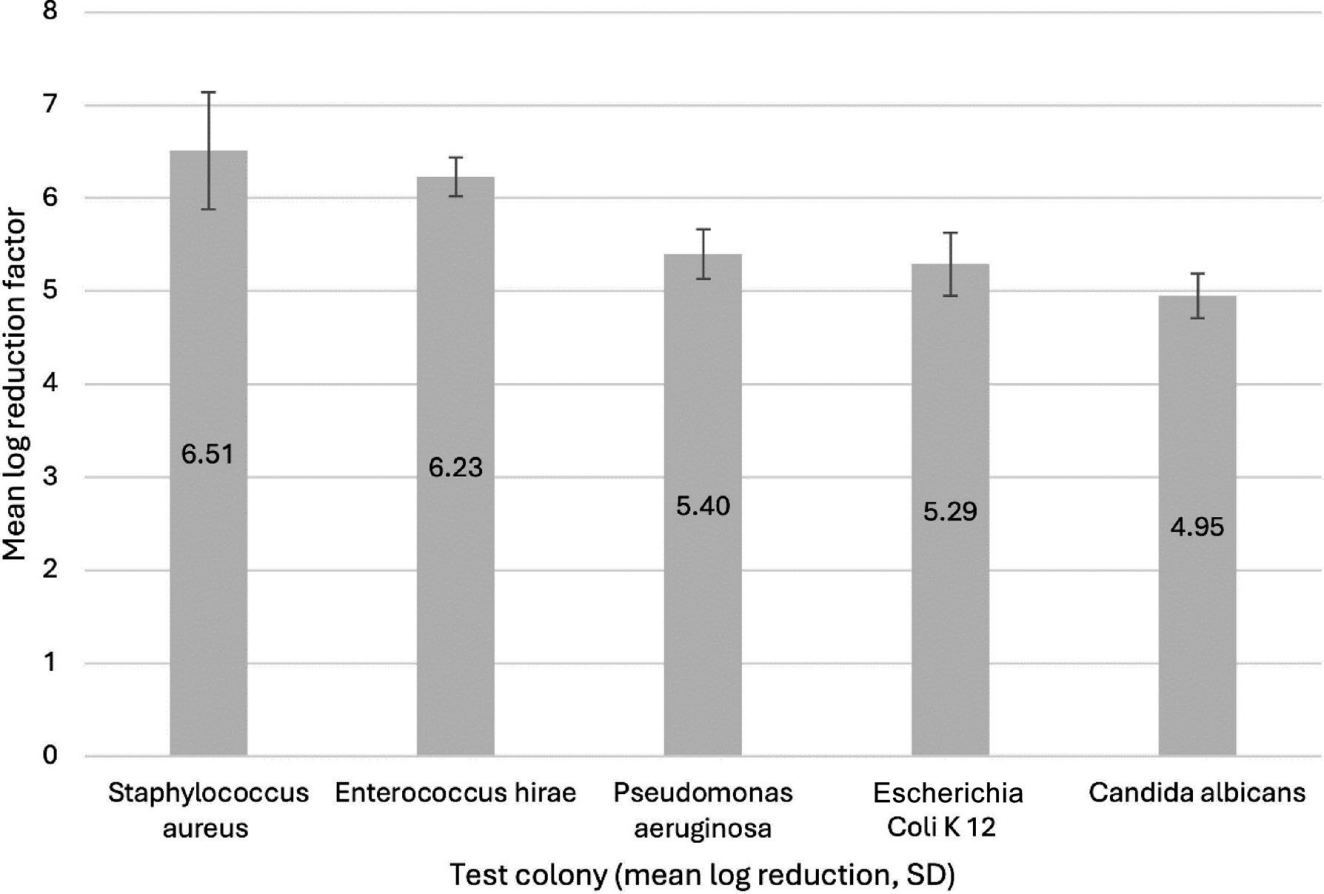
Graphical summary of the mean values of the log reduction factors including the standard deviation per test colony in descending order. SD = standard deviation.

The results indicate that the CAP-A process is more effective against gram-positive bacteria than gram-negative bacteria (Table 3, Fig. 6). However, the initial bacterial count limits the final reduction result. There is complete inhibition. The actual efficacy would be higher, but cannot be determined because the initial bacterial count is completely eliminated. For this reason, the reduction factors for Gram-positive and Gram-negative cannot be compared.

## Discussion

Ultrasound imaging is one of the most widely used and fastest growing diagnostic tools globally. Medical diagnostic devices such as transvaginal ultrasound probes can become contaminated with severe pathogens like HPV, chlamydia, and MRSA after use, which can lead to nosocomial infections (NI)^16,18,31–34^. NIs are the most frequent adverse events worldwide. In Europe, approximately 4,100,000 patients contract NIs annually^35^, which is equivalent to 80,000 infected patients daily^36^. One in 18 patients in European hospitals suffers from NI^36^. A meta-analysis estimates that up to half of all infections are preventable (35–55%)^37^. A study commissioned by a national health authority (Scotland) showed that gynecology patients who undergo transvaginal scans have a 41% higher infection risk, and 26% are more likely to be prescribed antibiotics^38^.

The findings of this study have important clinical implications, particularly for reducing the risk of NIs associated with medical devices such as ultrasound probes. Studies have shown that current disinfection practices, including manual wiping and low-level disinfection, are insufficient to ensure sterility^21^. CAP-A, as demonstrated in this study, offers a promising alternative with high disinfection efficacy, including against difficult-to-eliminate pathogens such as Clostridioides difficile^6^. Furthermore, the ability of CAP-A to maintain material integrity while ensuring high levels of microbial reduction makes it a highly suitable method for use in clinical settings. Several studies have highlighted the need for improved infection control measures, particularly for thermolabile devices such as ultrasound probes, where traditional disinfection methods often fail^7,21^. Further, laboratory conditions in the present test setting might be equivalent to a standardized, closed-container disinfection process of CAP-A within a clinical everyday setting. Given this exclusion of human-based errors, the potential impact of the CAP-A method for the prevention of NIs is considerable.

The results of the CAP-A method show it´s highly effective disinfection effect on ultrasound probes. It has been demonstrated that the practical inhibition test with selected test germs (*Staphylococcus aureus, Enterococcus faecium, Enterococcus hirae, Escherichia coli, Pseudomonas aeruginosa* and *Clostridioides difficile*) results in the complete inactivation of all microorganisms of the respective test contaminations. The apparent log reduction factors show that the effectiveness of the examined process reaches asepsis of the treated surfaces. The state of "non-infectivity" is achieved when at least three log levels of an initial germ count are eliminated, representing the inactivation of 99.9% of the initial germs (= 1000-fold reduction)^30^. This requirement is more than fulfilled by the application of the examined plasma disinfection process. Therefore, not only the state of "non-infectivity" is given, but it also results in a significantly increased safety of the disinfection result. The real microbiocidal potency is even higher. Since the initial germ count of the test contamination is limited, the resulting reduction performance is greater than the initial germ count in the case of complete inhibition.

The method could act as a promising decisive factor against the spread of NIs mentioned beforehand. CAP-A as a highly effective disinfection method relates to bactericidal, mycobactericidal, fungicidal, and virucidal organisms^38^. Further it is assumed from a microbiological perspective, that the inactivation of Clostridioides difficile guaranties the coverage of both enveloped and non-enveloped viruses. As the microbicidal test includes *Clostridioides difficile* bacterial endospores are also considered. The bacterial endospores represent the most tenacious known microorganisms. These pathogens pose a particular problem in hospital hygiene since most disinfectants have a gap in effectiveness against bacterial endospores (e.g., alcohols, phenols, quaternary ammonium compounds). Effectiveness is only achieved with corrosive chemicals as active ingredients: disinfection effects are known for hydrogen peroxide (> 30%), chlorine (> 1000 mg/l), chlorine dioxide (> 1000 mg/l) and other oxidizing agents^39^. However, these substances limit their use due to their strong chemical reactivity and corrosive-oxidative effect on the surfaces to be disinfected. This means that in everyday life, these disinfection chemicals can only be used indicatively, and the ongoing disinfection is still carried out with alcohols, phenols, and quaternary ammonium compounds (usually in the form of wipes soaked with active ingredients). Hence the presented method shows promising potential for reducing NIs and increasing patient safety^27,40^.

It is suitable for meeting the requirements of automated highly effective disinfection systems with standardized, reproducible processes and providing less operator dependency and greater ease through automation. It should be noted that the process using operating water, air oxygen, and dielectrically hindered discharges (plasma reaction) achieves a clear microbicidal efficacy while coming without hazardous substances and with minimal energy consumption (40 watts).

Another possible physical approach to disinfecting thermolabile medical devices, such as transvaginal ultrasound probes, is the use of UV light. However, effective UV disinfection requires uniform irradiation of the entire probe surface. Due to the complex geometry of the probes, including undercuts, folds and holes, shadows can form that affect the complete inactivation of microorganisms^41^. This is a major limitation compared to the CAP-A method, which can effectively reach hard-to-reach areas. Hydrogen peroxide-based systems are effective but can be corrosive and require longer exposure times, which may not be suitable for sensitive medical devices^42^. Alcohol-based disinfectants are widely used but have limitations in consistently inactivating all pathogens, particularly those that form biofilms or spores^21^. CAP-A, on the other hand, provides a non-thermal, chemical-free solution that is effective against a wide range of pathogens, including difficult-to-eliminate spores such as Clostridioides difficile^39^. CAP-A's ability to disinfect without direct contact and without leaving harmful residues makes it a promising alternative, particularly for thermolabile medical devices.

For reasons of feasibility and use of high-priced medical products, a compromise between the gap in effectiveness and the probability of the necessity for disinfection against endospore-forming pathogens had to be made so far. This resulted in sporicidal disinfectants being used indicatively, although the basic requirement of hygiene is primary prevention. In general, oxidative disinfection is only carried out when an indicator case occurs, and the gap in effectiveness of the other disinfectants for daily use had to be accepted. The results show that the examined process, besides the clear disinfection effect, now also closes the existing gap in effectiveness of sporicidal and simultaneously achieves the required safety in the form of 4.62 log levels. Against native microorganisms (microorganisms except for bacterial endospore formers), the process shows an average log reduction factor of > 5.8 log levels.

## Conclusion

In conclusion, the initial requirements for an effective disinfection process for thermolabile medical products (effectiveness, application safety, material compatibility, environmental friendliness) are met by the examined indirect method of CAP-A. Furthermore, the sporicidal against bacterial endospores should be noted, which is capable of closing a known gap in effectiveness of standard disinfection, also with regard to the reprocessing of thermolabile medical products. Finally, the discussed method increases patient safety through an automatable and thus validatable disinfection process. In addition to the outlined patients benefits the use of the investigated method of CAP-A leads to an increase in workplace safety. No measures are required to minimize the risk of unintended reactions from the use of hydrogen peroxide in the conventional disinfection of heat-sensitive medical devices^42^. In conclusion, a medical product based on CAP-A might be implemented in practice to enhance solving a globally existing problem for the hygienic treatment of thermolabile medical devices. The developed and analyzed test procedure could contribute to the achievement of selected United Nations Sustainable Development Goals (SDGs). The prevention of NI directly achieves SDG 3 (good health and well-being) and SDG 9 (build resilient infrastructure, promote inclusive and sustainable industrialization, and foster innovation).

## Limitations and outlook

While the method of CAP-A has shown promising efficacy in terms of microbial reduction and material compatibility, several limitations must be considered. A more thorough cost-effectiveness comparison with existing disinfection methods is warranted. Such an analysis would be essential to understand the wider applicability of CAP-A in clinical practice, particularly in terms of operational costs and resource utilization. Future research will aim to include this analysis to further evaluate the feasibility of CAP-A for widespread use in clinical settings.

In addition, the ERDF funding limited the number of laboratory measurements that could be performed, and most reactive species in the process of CAP-A are short-lived, with lifetimes in the millisecond range. Due to their transient nature, they cannot be analytically separated or measured by conventional methods, which limits the accurate characterization of plasma-induced microbial inactivation. In addition, increasing the sample size in future experiments would help control for theoretically conceivable biases due to measurement error. Replication in certified, multi-center testing laboratories could further reduce measurement bias and empirically support previous findings.

## Data Availability

All data generated or analyzed during this study are provided within the manuscript itself.
